# Text-Book of Nervous Diseases

**Published:** 1898-01

**Authors:** 


Text-Book of Nervous Diseases. By Charles L. Dana, A. M., M. D., New York. Fourth revised edition. One volume, octavo, 640 pages. Illustrated. New York: William Wood & Company. 1897.

The earlier editions of Dana's text-book of nervous diseases have been thoroughly reviewed in the Journal, so that the present edition needs no word of recommendation or introduction to the profession. This edition has been changed in some respects, some of the chapters entirely rewritten, as those on acute encephalitis, multiple sclerosis and combined sclerosis ; also the chapters on neurasthenia, while others have been rearranged and modified. The chapters on the microscopic anatomy of the nervous system and on the anatomy of the spinal cord and brain are practically new and have been made to conform more completely with present views regarding the importance of the *neuron*. Some new illustrations have been inserted while others have been removed, and yet the volume is not more bulky than the third edition.

The last edition upholds the reputation of the former editions as being the leading American text-book on nervous diseases.

The typography, under the supervision of Wm. Wood & Co., is of the best. W. C. K.

